# 4101 Creating a Culturally Sensitive Report Card for African American (AA) Kidney Transplant Candidates

**DOI:** 10.1017/cts.2020.193

**Published:** 2020-07-29

**Authors:** Warren McKinney, Marilyn J. Bruin, Sauman Chu, Bertram L. Kasiske, Ajay K. Israni

**Affiliations:** 1 University of Minnesota CTSI; 2 University of Minnesota - College of Design; 3 Hennepin Healthcare Research Institute

## Abstract

OBJECTIVES/GOALS: AA are over-represented on the waitlist for kidney transplant and are often unaware of how waitlist acceptance practices differ across transplant programs and influence access to transplant. We will develop a culturally sensitive transplant program report card to communicate these variations. METHODS/STUDY POPULATION: Scientific Registry of Transplant Recipients (SRTR) data will be used to identity clinical factors strongly associated with AA access to transplant. Interviews and focus groups with AA kidney transplant candidates and their families will collect feedback on the SRTR report card and inform the development of the culturally sensitive report card. Additional focus groups will evaluate its effect on knowledge and medical decision making. We will collaborate with the stakeholders, including AA transplant candidates and their families, transplant programs, SRTR, and providers, to identify strategies to disseminate the report card in the AA community RESULTS/ANTICIPATED RESULTS: To date, no investigation has systematically collected feedback on the SRTR transplant program report card from AA candidates to ensure that the tool is accessible and effective in the AA community. We hypothesize that a culturally sensitive report card will improve AA candidates’ knowledge of program factors that impact access to transplant and enable informed decisions about where they pursue a transplant evaluation. The results of this study have the potential to change how AA patients are counselled while seeking transplantation. DISCUSSION/SIGNIFICANCE OF IMPACT: A culturally sensitive report card can reach more AA patients and enable more informed decision making by providing education about differences in transplant programs that may impact their access to transplant. In the future, we will design a trial to evaluate the prototype.

